# Generative Adversarial Networks and Mixture Density Networks-Based Inverse Modeling for Microstructural Materials Design

**DOI:** 10.1007/s40192-022-00285-0

**Published:** 2022-11-08

**Authors:** Yuwei Mao, Zijiang Yang, Dipendra Jha, Arindam Paul, Wei-keng Liao, Alok Choudhary, Ankit Agrawal

**Affiliations:** Department of Electrical and Computer Engineering, Northwestern University, Evanston, IL USA

## Abstract

There are two broad modeling paradigms in scientific applications: forward and inverse. While forward modeling estimates the observations based on known causes, inverse modeling attempts to infer the causes given the observations. Inverse problems are usually more critical as well as difficult in scientific applications as they seek to explore the causes that cannot be directly observed. Inverse problems are used extensively in various scientific fields, such as geophysics, health care and materials science. Exploring the relationships from properties to microstructures is one of the inverse problems in material science. It is challenging to solve the microstructure discovery inverse problem, because it usually needs to learn a one-to-many nonlinear mapping. Given a target property, there are multiple different microstructures that exhibit the target property, and their discovery also requires significant computing time. Further, microstructure discovery becomes even more difficult because the dimension of properties (input) is much lower than that of microstructures (output). In this work, we propose a framework consisting of generative adversarial networks and mixture density networks for inverse modeling of structure–property linkages in materials, i.e., microstructure discovery for a given property. The results demonstrate that compared to baseline methods, the proposed framework can overcome the above-mentioned challenges and discover multiple promising solutions in an efficient manner.

## Introduction

Understanding the relationships between processing, structure, properties, and performance (PSPP) [1, 2] is critical in material science. In general, there are two broad modeling paradigms: forward and inverse. Forward modeling is to predict the effects or results given a set of known causes, e.g., exploring the relationships from processing to performance in materials. As different sets of inputs might cause the same result, forward modeling usually learns a many-to-one mapping. Forward modeling has been widely studied in various fields of machine learning, such as object detection [3, 4], image segmentation [5, 6], machine translation [7, 8] and some prediction tasks in scientific computing [9–16]. Inverse modeling is the process to infer the causes based on results or observations, e.g., exploring the relationships from performance to processing in materials. Inverse problems are one of the most important problems in science as they can help us understand the unknown causes leading to the observations. Thus, it is extensively used in various scientific fields, such as geophysics, health care and materials science [17–24]. Discovering microstructures that exhibit given properties is one of the inverse problems focused on structure–property linkage in materials, which is explored in this work. Variation in microstructure leads to a wide range of material properties, which in turn impacts the performance. Thus, inferring possible microstructures for a given property can help domain scientists improve the materials’ performance and accelerate materials discovery, and design. Traditional approaches [25, 26] for inverse modeling mainly rely on human analysis and experiments, which are extremely expensive in terms of cost and time. With the availability of large amounts of reliable data, data-driven methods have been tried to solve inverse problems. However, there are still many challenges for inverse modeling. The challenges for inverse modeling are threefold: (1) Inverse modeling usually requires learning a one-to-many nonlinear mapping. Because it is possible that different input combinations from many causal factors might cause the same output, there may be more than one microstructure that has a given property. This lack of uniqueness makes it difficult to train inverse models. (2) Inverse models usually need to learn a mapping from low-dimension inputs to high-dimension outputs, which means important missing information needs to be recovered from less informational inputs to produce high informational outputs. Thus, if the inverse model directly learns the mapping from inputs to outputs, the outputs might have limited diversity and only cover a small portion of real data distribution, especially when the difference of dimensionality between inputs and outputs is significant. In this work, the microstructures are represented by images, which are much more high-dimensional as compared to properties. (3) Traditional approaches for inverse modeling usually involve an iterative learning process, such as optimization, so that optimal or near-optimal solution can gradually be achieved by minimizing the error between candidate solution and target. However, due to the fact that the space of all possible causal factors can be extremely large, inverse modeling requires significant computing time. To overcome the above challenges, we propose a framework that combines generative adversarial networks (GAN) [27] and mixture density networks (MDN) [28] for inverse modeling. More specifically, a GAN is first trained so that the high-dimensional (i.e., high-resolution) microstructure image *x* can be represented by low-dimensional latent variable vector *z*. Then, we can utilize MDN, a neural network attempting to learn one-to-many nonlinear mapping (i.e., address challenge 1), to model the mapping from image property *y* to latent variable vector *z* instead of directly mapping from image property *y* to image *x*. Because latent variable vector *z* has similar dimensionality as the image property *y*, it is easier and more stable to train the MDN by using latent variable vector *z* as an immediate representation of image *x* (i.e., address challenge 2). Also, it is expected to increase the diversity of the outputs of the inverse model to cover a wider range of real data distribution. After the proposed framework is well trained, given a desired image property *y*, the MDN can produce various sets of latent variable vector *z*, which can be further used by GAN to generate corresponding images *x* to solve the inverse problem. Because the proposed framework is based on deep learning, it only requires one forward pass to produce various predictions, which means it can quickly produce possible solutions using modern computation resources (i.e., address challenge 3). We apply the proposed framework on a materials science inverse problem where microstructure images *x* need to be designed given a desired material’s optical absorption property value *y*. Three baseline methods are used to evaluate the performance of the proposed framework: (1) Optimization-based inverse modeling method; (2) Deep learning-based inverse modeling method that directly maps from material’s optical absorption property value *y* to microstructure images *x*; (3) The third baseline combines traditional dimensionality reduction, such as principal component analysis (PCA), and MDN to illustrate the advantage of using GAN. Compared with baseline methods, the results show that the proposed framework can not only generate solutions with properties closer to the target properties, but also produce more candidate solutions in an efficient manner. A conference version of this work appeared in [29], and the current article significantly expands on the conference paper with more background and details on the framework, subsequent analysis of results as well as significant insights and discussion.

## Materials and Methods

### Inverse Modeling

As described previously, modeling in science can be categorized into forward modeling and inverse modeling. Forward modeling is to predict the responses given a set of causal factors, which usually is a many-to-one mapping. In other words, the same observation could be produced by different causal factors. The problem can be formulated as $o=F(i)$ where *F* is the forward model, *i* is the vector of causal factors and *o* is the response. On the other hand, inverse modeling is to calculate from a set of observations the causal factors that produced them, which usually is a one-to-many mapping. It can be formulated as $i=G(o)$ where *G* is the inverse model and $G=F^{-1}$. Inverse modeling is one of the most important problems in science, because it can explore the causal factors that cannot be observed directly.

### Microstructural Materials Design

Microstructural materials design is one of the inverse problems in the field of materials science. It is the process to design materials microstructure to achieve a desired property of the resulting material. Microstructural materials design has revolutionarily changed the way to discover and design advanced materials [30]. In this work, we focus on the design of microstructure images with desired optical absorption property. Optical absorption is defined as the ability of the material to convert absorbed light into another energy form such as heat. Materials with high optical absorption properties can be used in solar cell design.

### Related Work

Inverse modeling, especially in the field of materials science, is usually developed based on either a forward model or an optimization method. In [31], forward models are trained and are subsequently used to scan millions of ternary compositions to screen for possible stable compounds. Forward models, as discriminative models, are incapable to generate new data. Thus, this method requires a significantly large amount of candidate data to be evaluated, and it cannot guarantee a possible solution existed in the candidate data. Therefore, the optimization method is commonly used for inverse modeling since it can usually produce possible solutions. In [18, 19], a framework combining a GAN model and an optimization method is used to design microstructure images with optimal material’s property. In [32], the desired property is achieved by optimizing the hierarchical motif-based topological fingerprints, which are used to reconstruct the molecular structures. However, optimization-based method can be very time-consuming, and it can only produce a limited number of solutions for the inverse problem.

Recently, deep learning has been used to solve inverse problems in several fields, and it can produce various possible solutions efficiently. For example, deep learning techniques have been actively researched for tomographic imaging, especially in the context of biomedicine, with impressive results and great potential [33]. In [34], authors use the deep residual learning for model-based iterative reconstruction. In [35], a model integrating mixture density networks and variational autoencoder is developed to produce an alloy composition given a partial phase diagram. However, the current study is different and more challenging compared to [35], because the microstructure images we aim to produce have much more degrees of freedom (i.e., high-resolution images) than alloy composition (i.e., a few numerical values) and our input material’s property (i.e., a float number) contains much less information than a partial phase diagram (i.e., a matrix).

In computer vision, [36, 37] implement GAN to generate images based on the description of the image. The differences between [36, 37] and our work are twofold. First, the image description (i.e., a sentence) contains much more information about the image than the material’s property. Second, the scenario can be very different due to the possibly higher variability across microstructure images compared to retinal images. [36, 37] pay more attention to local objects described in the image. For example, when the description is “this small bird has a pink breast and crown, and black primaries and secondaries,” the generated image is considered as successful as long as the image contains the bird with described characteristics, and the location of the bird and the surroundings are less important. However, in other scientific fields, such as materials science, it is crucial to capture the global characteristics of the image, because a small change in any location of the microstructure image might significantly affect its property. For example in Fig. 3a, although five microstructure images are visually similar, the difference of volume fraction (i.e., ratio between white and black materials) and spatial distribution of materials (i.e., spatial distribution of white and black materials) results in a significant difference in material’s property.

### Generative Adversarial Networks

Generative adversarial networks (GAN) [27] are a deep learning technique that originated from game theory. GAN consists of two components: generator and discriminator. Specifically, generator *G*(*z*) produces samples $x_G$ from latent variable vector *z* to approximate samples $x_{data}$ from real dataset, while discriminator *D*(*x*) distinguishes the generated samples $x_G$ from real samples $x_{data}$. Essentially, GAN is defined as a minimax game, which can be formulated as the following equation,

1 $$\begin{aligned} \min _{G} \max _{D} V(D,G) = {\mathbb {E}}_{x\sim {p_{data}(x)}}[\log {D({{\textbf {x}}})}] + {\mathbb {E}}_{{{\textbf {z}}}\sim {p_z(z)}}[\log (1-D(G({{\textbf {z}}})))] \end{aligned}$$

where ${p_z(z)}$ is the prior distribution of the latent variable vector *z*, and ${p_{data}(x)}$ is the distribution of the real data $x_{data}$. This minimax game would eventually lead to a convergence where the generator can generate data similar to real data that cannot be distinguished by the discriminator.

### Mixture Density Networks

Mixture density network (MDN) [28] is a type of neural network attempting to address the inverse problem. Instead of predicting a single value, the goal of MDN is to predict an entire probability distribution for the output (i.e., latent variable vector *z*) based on input (i.e., optical absorption property value *y*). MDN is usually constructed by a neural network to parameterize a mixture model consisting of some predefined distributions. Generally, Gaussian distribution is used, and the output is modeled as a conditional probability *P*(*z*|*y*) calculated by a weighted sum of *K* Gaussian distributions $\phi$ with different means $\mu$ and standard deviations $\sigma$, which can be defined as follows.

2 $$\begin{aligned} P(z|y) = \sum _{k=1}^{K} \pi _{k}(y)\phi (z|\mu _k(y),\sigma _k(y)), \sum _{k=1}^{K} \pi _{k}(y)=1 \end{aligned}$$

where *y* and *z* are inputs and outputs, respectively. $\pi _k$, $\mu _k$ and $\sigma _k$ are the mixing coefficient, mean and standard deviation of the $k^{th}$ Gaussian distribution, respectively. The network is updated by minimizing the logarithm of the likelihood of the distribution versus the training data,

3 $$\begin{aligned}&\min L(w) = \frac{-1}{N}\sum _{n=1}^{N}\nonumber \\&\log \left( \sum _{k}\pi _{k}(y_n, w)\phi (z_n|\mu _k(y_n,w),\sigma _k(y_n,w))\right) \end{aligned}$$

where *N* is the batch size, *w* is the weights of the MDN, $y_n$ is the $n^{th}$ instance in a batch and $z_n$ is the corresponding label.

### Densely Connected Neural Network

Due to the flexible architecture and a large number of hyperparameters, deep learning models have striking learning capability. However, when the depth of a deep learning model reaches a certain point, it usually encounters the vanishing gradient problem where the accuracy becomes saturated and degrades rapidly as the depth of the model increases. To avoid this problem, [3] introduced residual networks (ResNet) where the networks have a so-called identity shortcut connection to skip one or more stacked layers. Later, [38] introduced the concept of densely connected convolutional network (DenseNet). In DenseNet, each layer is connected to every other layer in a feed-forward fashion. In other words, each layer obtains additional inputs from all preceding layers to calculate its outputs. DenseNet has several advantages that can alleviate the vanishing gradient problem and strengthen feature reuse. Thus, dense connections are used in the architecture of MDN.

### Proposed Method

**Fig. 1:**
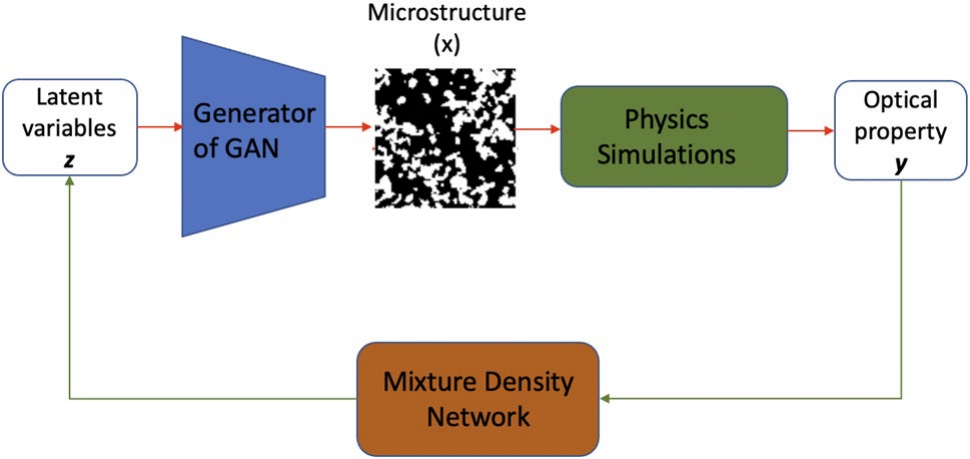
The flowchart of the proposed method. The red path shows the flow of data generation, and the green path represents the training of the proposed densely connected MDN

The flowchart of the proposed method is shown in Fig. 1. The proposed method consists of GAN and MDN where GAN is used to obtain the low-dimensional design representations (i.e., latent variable vector) of the microstructure images, and the MDN models are used to obtain the mapping between latent variable vector and design objective (i.e., material’s optical absorption property). We utilize the GAN trained in [18, 19], which is a fully convolutional neural network where both generator and discriminator have five layers. Specifically, each generator layer is a deconvolutional layer attached with batch normalization (BN) operation and rectified linear unit (ReLU) activation, except the last layer which uses a tanh activation function to produce the bounded pixel values for generated images. The number of filters in the five deconvolutional layers is 128, 64, 32, 16 and 1, respectively. Each discriminator layer consists of a convolutional layer, BN operation and leaky rectified linear unit activation, except the last layer which uses a sigmoid activation function to predict whether the image is fake or real. The number of filters in the five convolutional layers is 16, 32, 64, 128 and 1, respectively. For both convolutional and deconvolutional layers, the filter size is $4\times {4}$ with stride 2, except the last convolutional layer in the discriminator where the filter size is the same as the size of its input feature maps to produce probabilities. In order to avoid model collapse and impose morphology constraints of the generated images, model collapse loss and style transfer loss are added in addition to adversarial loss (see [18, 19] for details about customized loss function).

**Fig. 2:**
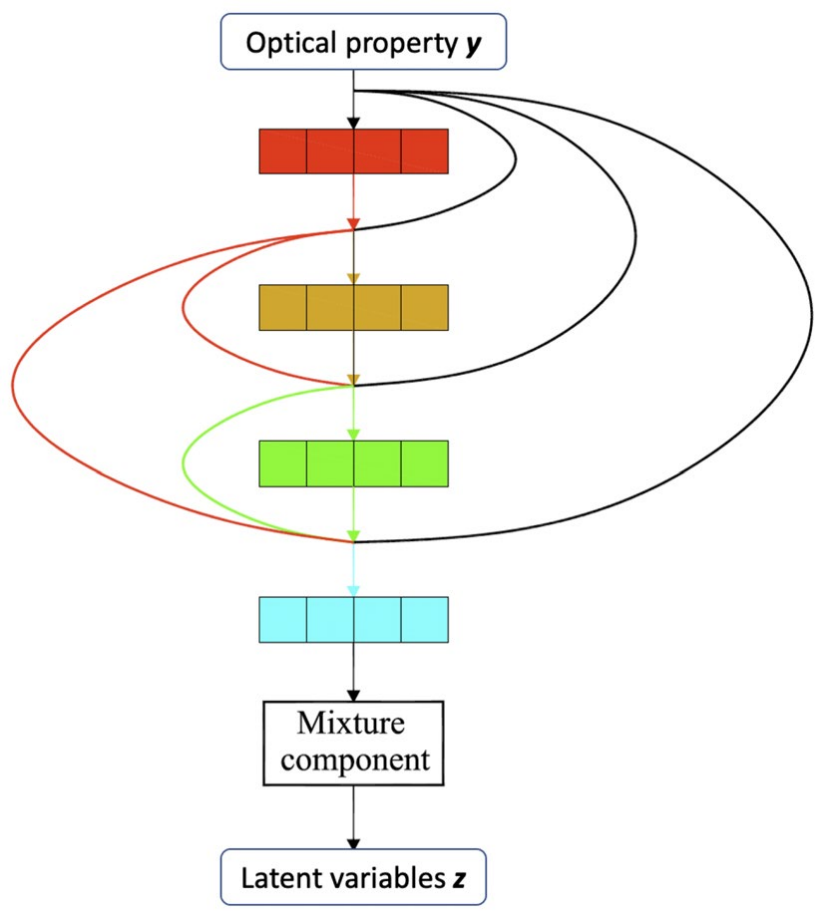
The architecture of the proposed MDN. MDN is constructed by four densely connected fully connected layers and a mixture component that models a mixture of Gaussian distributions

As shown in Fig. 2, MDN is constructed by four densely connected fully connected layers and a mixture component that models a mixture of Gaussian distributions. Each densely connected fully connected layer has 16 neurons followed by BN operation and ReLU activation, and each layer is connected with subsequent layers. In other words, each layer obtains additional inputs from all preceding layers (including the input layer) to calculate its outputs. The mixture component contains a mixture of 40 multivariate Gaussian distributions, which are parameterized by a fully connected layer. Assuming *M* denotes the dimension of the output (i.e., the dimension of latent variable vector of GAN), each multivariate Gaussian distribution needs one neuron for its mixing coefficient, two neurons for the mean and standard deviation of each dimension of latent variable vector. In particular, a linear activation function is used for the neurons computing mixing coefficients and means, while the exponential linear unit (ELU) [39] is used for the neurons calculating standard deviations. Thus, the number of neurons in the mixture component is computed as follows,

4 $$\begin{aligned} N = K\times {(1+2\times {M})} \end{aligned}$$

where *N* is the number of neurons of the mixture component, and *K* is the number of multivariate Gaussian distributions used in MDN ($K=40$ in the proposed model). During prediction time, we randomly select a distribution based on its mixture coefficient to sample the latent variable vectors. For MDN training, we use equation 3 as the loss function. Adam optimizer [40] with a batch size of 128 and learning rate of 0.001 is used. Early stopping with a patience of 50 is applied so that the training process is terminated when the loss function on the validation set does not improve for 50 epochs.

## Results

### Datasets and Error Metric

In [18, 19], the GAN is trained on 5000 synthetic microstructure images, which are created using Gaussian Random Field (GRF) method. The parameters in GRF (i.e., mean, standard deviation, and volume fraction) are carefully controlled to produce microstructures that cover the vast space of compositional and dispersive patterns, which corresponds to different processing conditions of the same material system. Then, the GAN and physics simulation in [18, 19, 41] are used to generate two datasets used in this work. The size of microstructure image *x* and the latent variable vector *z* of one dataset are $96\times {96}$ and $3\times {3}$ (referred as Data-I), and of the other are $64\times {64}$ and $2\times {2}$ (referred as Data-II), respectively. More specifically, the latent variable vector *z* is randomly generated and passed through the generator to generate the corresponding microstructure *x*. Then, the optical absorption property *y* of the generated microstructure is simulated using physics simulation (i.e., the rigorous coupled wave analysis [41]). Around 25000 data points are generated for each dataset, and the optical absorption property is distributed from 0.55 to 0.75. Thus, they could be used to train the proposed densely connected MDN to learn the mapping between latent variable vector *z* and optical absorption property *y*. Particularly, 70% of each dataset is used as the training set and the rest is used as the validation set to select the optimal hyperparameters of neural networks.

Residual error percentage (REP) is used to evaluate the performance of models, which is defined as equation 5,

5 $$\begin{aligned} REP = \frac{\mid {\hat{y}} - y \mid }{y} \times 100 \% \end{aligned}$$

where ${\hat{y}}$ and *y* are the optical absorption property of generated microstructure and target optical absorption property, respectively.

### Baselines

An optimization-based inverse modeling method, a deep learning method based on MDN without GAN, and a method combining PCA (which is used to replace GAN) and MDN are selected as baseline methods in this work.

**Optimization-Based Inverse Modeling:** The inverse modeling method based on optimization in [18, 19] is considered as a baseline method in this work. More specifically, for each target optical absorption property, 250 sample pairs (*z*, *y*) are sampled in the design representations (i.e., latent variable vector of GAN) space to create the response surface between latent variable vector and materials optical absorption property. Then, metamodel-based Bayesian optimization is conducted to iteratively explore the next potentially optimal design point. A total of 400 iterations of optimization are conducted after initial sampling of 250 points to ensure the convergence of the optimization process.

**MDN-Based Deep Learning Inverse Modeling:** In order to illustrate that it is easier and more stable to learn the mapping from materials optical absorption property *y* to latent variable vector *z* of GAN instead of directly mapping from material’s optical absorption property *y* to microstructural images *x*, we use a deep learning inverse modeling solely based on MDN as another baseline. More specifically, MDN takes material’s optical absorption property *y* as input and directly produces microstructural images *x*. The MDN in this baseline is the same as the MDN in the proposed framework, except the number of neurons in the mixture component is different because each pixel in microstructure image *x* can be considered as one dimension of the output. Other hyperparameter settings and training strategy are the same as the proposed framework.

**PCA- and MDN-Based Inverse Modeling (Referred as PCA-MDN Method):** In order to illustrate the advantage of using GAN to obtain the low-dimensional design representations (i.e., latent variable vector) as compared to traditional dimensionality reduction methods, PCA is used to replace GAN and combined with MDN to produce microstructure images *x* given a desired materials optical absorption property *y*. More specifically, MDN takes material’s optical absorption property *y* as input and generates a reduced set of principal components, which is used by PCA to inversely transform to corresponding microstructure images *x*. The dimension of a reduced set of principal components is the same as the dimension of the latent variable vector in the proposed framework, which is 9 for Data-I and 4 for Data-II. MDN is also exactly the same as the MDN in the proposed framework.

### Results of Inverse Modeling

We select five target optical absorption properties (i.e., 0.55, 0.60, 0.65, 0.70 and 0.75) to cover the range of possible optical absorption properties. For each target optical absorption property, we use the proposed densely connected MDN to sample 30 latent variable vectors *z* where we randomly select a distribution based on its mixture coefficient to sample the latent variable vectors. Each latent variable vector *z* is then passed through GAN [18, 19] to generate microstructure images. Finally, their corresponding optical absorption property can be simulated by physics simulation [18, 19, 41] and compared with the target optical absorption property. The same evaluation strategy is used for the baseline methods, MDN-based deep learning inverse modeling, and PCA-MDN method. For the inverse problem, discovering the microstructure with property closest to the target property is the most important goal. Thus, min REP is the most important evaluation metric for inverse models. The ability to discover multiple and diverse microstructures is also important for microstructure discovery inverse problems. Average REP and standard deviation of REP could evaluate the performance of multiple microstructures that provided properties close to target property.

**Fig. 3:**
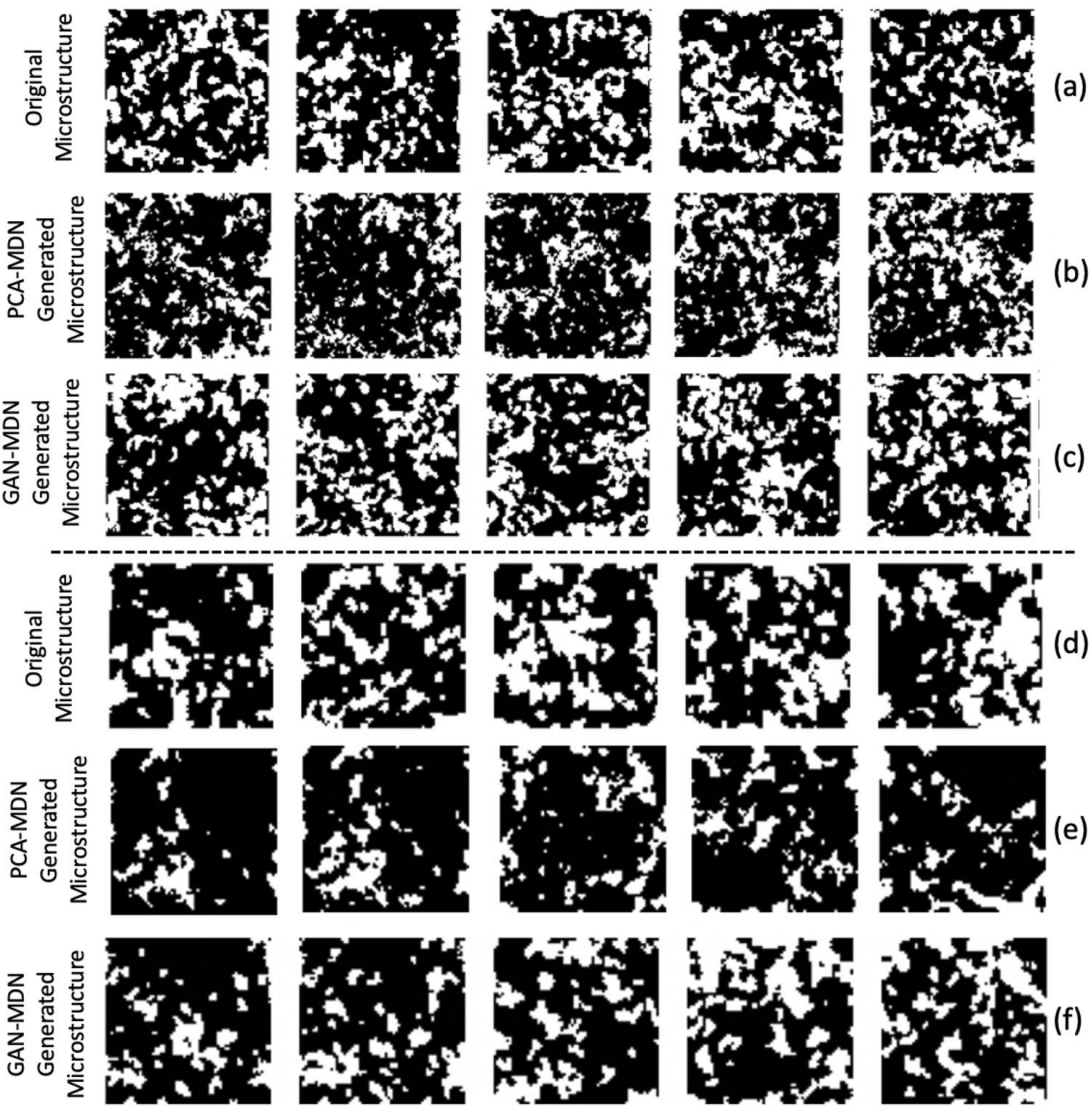
Examples of original microstructures and microstructures produced by the PCA-MDN approach and the proposed GAN-MDN approach for Data-I and Data-II. Row **a** and **d** are microstructures randomly selected in Data-I and Data-II. Row **b** and **e** are microstructures produced by PCA-MDN with minimum REP w.r.t. the target optical absorption properties. Row **c** and **f** are microstructures produced by the proposed GAN-MDN with minimum REP to the target optical absorption properties. The target optical absorption properties are 0.55, 0.60, 0.65, 0.70 and 0.75 from left to right

**Results on Data-I:** Table 1 shows the performance of the proposed framework on Data-I. We can observe that the min REP of the proposed method is lowest for most target properties and much less than 1%, which indicates that the proposed method can generate microstructures with optical absorption properties very close to the target property. Moreover, the average REPs of the proposed method are also the lowest for most target values, which indicates that the proposed method can generate multiple microstructures with properties close to the target property as compared to other methods. Figure 3a and c shows some examples of original microstructures and microstructures produced by the proposed GAN-MDN method that have the min REP w.r.t each target optical absorption property for Data-I. It shows that the proposed GAN-MDN method is capable of producing latent variable vectors *z* that generate visually similar microstructures as the original microstructures in the dataset. Further, it only takes around 10 s to produce the designed microstructural images.

**Table 1:** Performance of the GAN-MDN method, PCA-MDN-based baseline method, MDN-based deep learning inverse modeling baseline method and optimization-based inverse modeling baseline method on Data-I

Value	Min REP (%)	Average REP (%)	Standard deviation of REP (%)	Running time
**The GAN-MDN method**				
**0**.**55**	**0.65**	15.68	8.40	9.75 s
**0**.**60**	**0.18**	9.15	5.97	9.50 s
**0**.**65**	0.22	5.80	3.93	9.67 s
**0**.**70**	**0.13**	5.29	3.86	9.62 s
**0**.**75**	**0.20**	7.83	3.91	9.50 s
**Baseline: PCA-MDN method**				
**0**.**55**	5.05	17.67	7.84	7.22 s
**0**.**60**	0.50	10.89	6.48	7.30 s
**0**.**65**	**0.17**	5.92	4.00	7.20 s
**0**.**70**	0.40	8.81	5.27	7.20 s
**0**.**75**	2.95	18.34	5.54	7.36 s
**Baseline: MDN-based deep learning inverse modeling**				
**0**.**55**	0.84	9.07	3.14	175.27 s
**0**.**60**	4.70	14.40	4.08	187.86 s
**0**.**65**	9.35	20.04	4.06	177.60 s
**0**.**70**	12.29	25.18	4.21	147.23 s
**0**.**75**	17.73	26.81	3.55	178.70 s
**Baseline: Optimization-based inverse modeling**				
**0**.**55**	-	–	–	4.4 h
**0**.**60**	1.08	–	–	3.6 h
**0**.**65**	3.38	–	–	5.8 h
**0**.**70**	–	–	–	10.6 h
**0**.**75**	–	–	–	8.9 h

Bold values indicate the best values of the primary evaluation metric (min REP)

The results of the PCA-MDN method are also shown in Table 1. We can observe that the proposed method has significantly better min REP than the PCA-MDN method for low and high target optical absorption property values (i.e., 0.55, 0.70 and 0.75). This might indicate that this baseline method is incapable to capture all the significant information, so it fails to generate microstructures with property values close to the target property values since PCA can lose more information during the inverse transformation of the reduced set of principal components, i.e., latent variable vector *z* to microstructure images *x*, compared to GAN. Figure 3b shows some examples of microstructures produced by PCA-MDN for Data-I. Similar to the proposed method, it only takes a few seconds to produce microstructures.

Table 1 also shows the performance of MDN-based deep learning inverse modeling baseline method. The results show that both min REPs and average REPs are much higher than that of the proposed framework for most target values, and REP increases as the target optical absorption property increases. This is because this baseline method mainly produces microstructure images with low optical absorption properties. In other words, this baseline method focuses more on the low optical absorption property range of real data distribution. Thus, it fails to generate possible microstructure images when the target optical absorption property is high. As discussed in the introduction section, the significant difference of dimensionality between optical absorption property *y* and microstructure images *x* makes the training of the inverse model even more difficult. Thus, the diversity of the generated microstructure images is limited and only a small portion of real data distribution is covered by directly modeling the relationship between optical absorption property and microstructure images. In contrast, by using the latent variable vector *z* as the immediate representation of microstructure images *x*, the proposed framework provides diverse microstructure images along with the entire range of optical absorption properties. Although this baseline is also based on deep learning, it takes more time to produce microstructures compared to the proposed method since it directly maps optical absorption property *y* to microstructure images *x*.

**Fig. 4:**
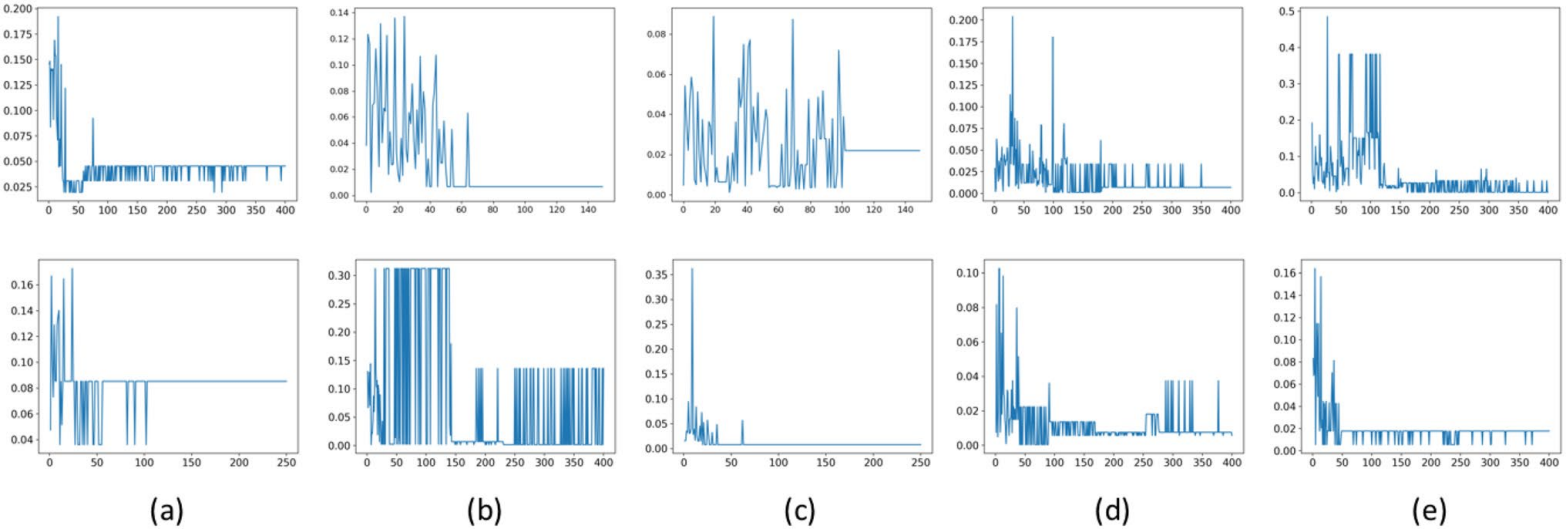
The microstructure optimization history for each target optical absorption property for Data-I (i.e., first row) and Data-II (i.e., second row). The x axis shows the iteration number, and the y axis shows the absolute REP between the target optical absorption property and the optical absorption property of sampled microstructure. The target optical absorption properties of each plots are 0.55 **a**, 0.60 **b**, 0.65 **c**, 0.70 **d** and 0.75 **e**

The performance of the optimization-based baseline method on Data-I is also listed in Table 1, and the first row in Fig. 4 shows the microstructure optimization history for each target optical absorption property. Since the optimization method can only produce one candidate microstructure, the average and standard deviation of REP are not applicable. For target properties 0.6 and 0.65, this baseline method reaches convergence around 65 and 105 epochs, respectively. However, this baseline method cannot converge when target properties are 0.55, 0.7 and 0.75. We can thus observe significant advantages of the proposed framework because the optimization-based method could not get comparable performance as the proposed method or even could not converge in some cases. The results indicate that optimization-based inverse modeling cannot successfully capture the relationship between latent variable vector *z* and optical absorption property *y* and is incapable to generate microstructures with desired property for all values. In addition, optimization-based inverse modeling can only produce a limited number of candidate microstructures due to the nature of the optimization method, while the proposed framework can sample as many candidate microstructures as the user wants. More importantly, it takes hours for optimization-based inverse modeling to optimize the microstructure for desired optical absorption property, while it only needs one forward pass for the proposed framework to produce microstructures, which only takes around 10 s using a Titan X GPU.

**Results on Data-II:** Table 2 shows the performance of the proposed framework on Data-II. The min REP and average REP for each target optical absorption property are extremely small, and the performance is comparable with that on Data-I. In addition, we can observe in Fig. 3d and f that the proposed framework can generate visually similar microstructures as microstructures in the dataset.

The results of the PCA-MDN method are also shown in Table 2. The results of the PCA-MDN method are comparable to the proposed method, and it achieves a better average REP in some cases. This might be because the microstructure image in Data-II is smaller than in Data-I so PCA is able to capture enough information. However, the min REP of the PCA-MDN method is significantly worse for some target properties, which might indicate it is not stable to use PCA to obtain low-dimensional design representations compared to GAN. Figure 3e shows some examples of microstructures produced by PCA-MDN for Data-II. In addition, similar to Data-I, it only takes a few seconds for the PCA-MDN method to produce microstructures.

Table 2 presents the performance of MDN-based deep learning inverse modeling method, and the performance is much worse than that of the proposed framework. Similar to its performance on Data-I, it again focuses more on the low optical absorption property range of real data distribution, but it covers a wider property range since it performs better on high property values on Data-II. This may be because the microstructure image in Data-II is smaller than that in Data-I, so it might be easier for MDN to directly map from optical absorption property *y* to microstructure image *x*. This observation also supports our conclusion that it is easier and more stable to train the MDN by using latent variable vector *z* as an immediate representation of image *x* since the performance of the proposed framework on two datasets is similar. Since microstructure images in Data-II are smaller, it takes less time to produce microstructures compared to running time on Data-I.

**Table 2:** Performance of the proposed method, PCA-MDN-based baseline method, MDN-based deep learning inverse modeling baseline method and optimization-based inverse modeling baseline method on Data-II

Value	Min REP (%)	Average REP (%)	Standard deviation of REP (%)	Running time
**The proposed method**				
**0**.**55**	**1.25**	16.19	8.96	9.67 s
**0**.**60**	0.70	10.99	7.93	9.74 s
**0**.**65**	**0.18**	7.65	5.64	9.57 s
**0**.**70**	**0.10**	5.00	4.61	9.68 s
**0**.**75**	**0.43**	6.18	3.51	9.60 s
**Baseline: PCA-MDN method**				
**0**.**55**	4.96	11.74	3.05	7.24 s
**0**.**60**	**0.07**	2.69	2.18	7.26 s
**0**.**65**	3.71	8.79	2.59	7.40 s
**0**.**70**	**0.10**	3.41	2.44	7.15 s
**0**.**75**	3.17	6.27	1.52	7.26 s
**Baseline: MDN-based deep learning inverse modeling**				
**0**.**55**	2.85	12.78	3.89	23.21 s
**0**.**60**	7.87	14.95	3.56	24.05 s
**0**.**65**	11.00	17.33	2.63	24.14 s
**0**.**70**	3.03	15.62	4.09	23.90 s
**0**.**75**	8.44	12.73	3.20	23.34 s
**Baseline: Optimization-based inverse modeling**				
**0**.**55**	15.51	−	−	5.8 h
**0**.**60**	−	−	−	12.1 h
**0**.**65**	1.21%	−	−	4.2 h
**0**.**70**	−	−	−	18.8 h
**0**.**75**	−	−	−	3.2 h

Bold values indicate the best values of the primary evaluation metric (min REP)

The second row in Fig. 4 shows the optimization history of the optimization-based baseline method on Data-II. We can observe that when the target properties are 0.55 and 0.65, the baseline method reaches convergence around 110 and 70 epochs, respectively. However, it fails to converge when target properties are 0.6, 0.7 and 0.75. The performance of the optimization-based inverse modeling method is also presented in Table 2. It shows that the performance of the proposed framework is much better than that of the optimization-based method. More importantly, it still takes hours for the optimization-based method to produce the solutions even though the dimension of the latent variable vector to be optimized for Data-II is smaller than that for Data-I, and it is much slower than the proposed framework.

### Exploration of Material Property

Exploration is as important as exploitation, since it can lead to materials discovery with enhanced material property. Table 3 and Table 4 show the performance of the proposed model and deep learning-based baseline methods in designing materials with optical absorption property out of the range of training set on the two datasets, respectively. More specifically, optical absorption property values of 0.53 and 0.77 are used as target values to design material microstructures. From the results, we can see that overall the proposed method is better and more consistent than baseline methods considering both property values for both datasets. The min REPs (especially on Data-II) show better ability of the proposed method to expand the range of material’s property of training set by generating microstructures with property slightly outside of training set range. Then, the proposed model can potentially be fine-tuned with extended training set to capture the new data distribution. The two steps can be iteratively repeated so that it provides an opportunity to explore materials with a wider range of properties.

**Table 3:** Performance of the proposed method, PCA-MDN-based baseline method and MDN-based deep learning inverse modeling baseline method on Data-I

Value	Min REP (%)	Average REP (%)	Standard deviation of REP (%)	Running time (s)
**The proposed method**				
**0**.**53**	1.43	16.66	9.72	9.70 s
**0**.**77**	0.78	10.78	5.73	9.48 s
**Baseline: PCA-MDN method**				
**0**.**53**	7.35	20.96	9.12	7.06 s
**0**.**77**	**0.30**	9.40	6.18	7.35 s
**Baseline: MDN-based deep learning inverse modeling**				
**0**.**53**	**0.44**	7.42	3.31	23.12 s
**0**.**77**	16.00	28.49	4.05	22.87 s

Bold values indicate the best values of the primary evaluation metric (min REP)

**Table 4:** Performance of the proposed method, PCA-MDN-based baseline method and MDN-based deep learning inverse modeling baseline method on Data-II

Value	Min REP (%)	Average REP (%)	Standard deviation of REP (%)	Running time (s)
**The proposed method**				
**0**.**53**	**0.38**	18.78	8.48	9.52 s
**0**.**77**	**0.50**	7.82	3.60	9.31 s
**Baseline: PCA-MDN method**				
**0**.**53**	25.33	33.32	3.63	6.96 s
**0**.**77**	6.28	10.66	2.20	7.05 s
**Baseline: MDN-based deep learning inverse modeling**				
**0**.**53**	12.49	19.02	3.77	23.51 s
**0**.**77**	16.15	25.27	3.26	23.04 s

Bold values indicate the best values of the primary evaluation metric (min REP)

## Discussion

The proposed framework can generate more candidate microstructures in an efficient manner for desired property, and it can be utilized in two main cases. (1) **Materials design:** It is crucial to produce various possible microstructures in materials design because there are many other unknown factors that might affect material’s property, such as human operation and manufacturing technology, which might result in the difference between the property of the designed microstructures and manufactured ones. In other words, more possible solutions for materials design provide more possibilities to design the materials with the desired property. Moreover, by investigating the similarity and differences of various candidate microstructures produced by the proposed framework, domain scientists can obtain more information about how to design the microstructures, which can help them to conduct experiments in a more informed way. (2) **Materials discovery:** It is difficult or even impossible in many cases to obtain a large reliable dataset using traditional time-consuming research approaches, such as experiments. Using the proposed framework, domain scientists can easily obtain a large number of microstructures with various property values. The huge dataset can provide an effective way to investigate the characteristics of microstructures with various properties. This can provide an opportunity to discover underlying characteristics of materials for property improvement, which can in turn lead to the discovery of new advanced materials. In the future, we will try to extend the proposed framework to other inverse problems in materials science (e.g., processing-structure linkage) and possibly other scientific fields.

## Data Availability

The datasets and source code used in this study are available at https://github.com/zyz293/GAN-MDN
